# Analysis of the spatial distribution of dengue cases in the city of Rio de Janeiro, 2011 and 2012

**DOI:** 10.11606/S1518-8787.2017051006239

**Published:** 2017-08-03

**Authors:** Silvia Carvalho, Mônica de Avelar Figueiredo Mafra Magalhães, Roberto de Andrade Medronho

**Affiliations:** I Centro de Informações Estratégicas em Vigilância em Saude. Subsecretaria de Vigilância em Saúde. Secretaria de Estado de Saúde do Rio de Janeiro. Rio de Janeiro, RJ, Brasil; II Instituto de Comunicação e Informação Científica e Tecnológica em Saúde. Fundação Oswaldo Cruz. Rio de Janeiro, RJ, Brasil; III Faculdade de Medicina. Universidade Federal do Rio de Janeiro. Rio de Janeiro, RJ, Brasil

**Keywords:** Dengue, epidemiology, Spatial Analysis, Geographic Information Systems, Health Information Systems, Dengue, epidemiologia, Análise Espacial, Sistemas de Informação Geográfica, Sistemas de Informação em Saúde

## Abstract

**OBJECTIVE:**

Analyze the spatial distribution of classical dengue and severe dengue cases in the city of Rio de Janeiro.

**METHODS:**

Exploratory study, considering cases of classical dengue and severe dengue with laboratory confirmation of the infection in the city of Rio de Janeiro during the years 2011/2012. The georeferencing technique was applied for the cases notified in the Notification Increase Information System in the period of 2011 and 2012. For this process, the fields “street” and “number” were used. The ArcGis10 program’s Geocoding tool’s automatic process was performed. The spatial analysis was done through the kernel density estimator.

**RESULTS:**

Kernel density pointed out hotspots for classic dengue that did not coincide geographically with severe dengue and were in or near favelas. The kernel ratio did not show a notable change in the spatial distribution pattern observed in the kernel density analysis. The georeferencing process showed a loss of 41% of classic dengue registries and 17% of severe dengue registries due to the address in the Notification Increase Information System form.

**CONCLUSIONS:**

The hotspots near the favelas suggest that the social vulnerability of these localities can be an influencing factor for the occurrence of this aggravation since there is a deficiency of the supply and access to essential goods and services for the population. To reduce this vulnerability, interventions must be related to macroeconomic policies.

## RESUMO

**OBJETIVO:**

Analisar a distribuição espacial dos casos de dengue clássico e dengue grave no município do Rio de Janeiro.

**MÉTODOS:**

Estudo exploratório, considerando casos de dengue clássico e de dengue grave com comprovação laboratorial da infecção, ocorridos no município do Rio de Janeiro nos anos de 2011/2012. Foi aplicada a técnica de georreferenciamento dos casos notificados no Sistema de Informação de Agravos de Notificação, no período de 2011 e 2012. Para esse processo, utilizaram-se os campos “logradouro” e “número”. Foi realizado o processo automático da ferramenta *Geocoding* do programa *ArcGis10.* A análise espacial foi feita a partir do estimador de densidade Kernel.

**RESULTADOS:**

A densidade de Kernel apontou áreas quentes para dengue clássico não coincidente geograficamente a dengue grave, estando localizadas dentro ou próximas de favelas. O cálculo da razão de Kernel não apresentou modificação significativa no padrão de distribuição espacial observados na análise da densidade de Kernel. O processo de georreferenciamento mostrou perda de 41% dos registros de dengue clássico e 17% de dengue grave devido ao endereçamento da ficha do Sistema de Informação de Agravos de Notificação.

**CONCLUSÕES:**

As áreas quentes próximas às favelas sugerem que a vulnerabilidade social existente nessas localidades pode ser um fator de influência para a ocorrência desse agravo, uma vez que há deficiência da oferta e acesso a bens e serviços essenciais para a população. Para diminuir essa vulnerabilidade, as intervenções devem estar relacionadas a políticas macroeconômicas.

## INTRODUCTION

Dengue is one of the most important tropical diseases in the world due to its high incidence and potential for dissemination. It is closely related to climatic variables and the political, economic and socio-environmental conditions that favor the proliferation of its vector^22^. Some chronic conditions are also identified as possible individual risk factors for the occurrence of SD^11^.

It is caused by an arbovirus of the genus Flavivirus (serotypes 1, 2, 3 and 4) and transmitted by the *Aedes Aegypti* mosquito^6^ ^,^ ^17^.

It is known that early and appropriate diagnosis and treatment are essential to reduce the lethality of the disease. Currently, the World Health Organization (WHO) adopts a simpler classification of dengue cases: non-severe dengue or severe dengue^21^.

The serotype DENV-1 was detected in the state of Rio de Janeiro in 1986. Since then, intense transmission of the disease has been observed. With the emergence of DENV-2, in 2008, there was an epidemic with the highest number of cases ever in the state^a^.

In this context, the city of Rio de Janeiro is an important scenario to understand the factors that interfere in the transmission dynamics and worsening of the disease, since it has considerable socioeconomic and demographic differences among its administrative regions and it also had the highest percentage of notified cases in the state.

From the above, this study proposes to analyze the spatial distribution of classical dengue and severe dengue cases in the city of Rio de Janeiro, in order to contribute to the decision-making process to effectively control the disease in the city.

## METHODS

In this ecological study, the spatial distribution of classical dengue fever (CD) cases and severe dengue (SD) cases in the city of Rio de Janeiro during the years 2011 and 2012 was carried out only with cases that had laboratory confirmation of the infection. We opted to utilize only cases confirmed by a laboratory so that there wouldn’t be any doubts regarding the diagnoses.

The city of Rio de Janeiro is composed of 160 neighborhoods distributed in 33 administrative regions (RA). It has an estimated population of 6,497,728 people in a territorial extension of 122,456.01 km^2^, encompassed by residential areas - outside and within subnormal clusters (favelas), commercial sectors, parks, beaches, lakes and the Atlantic Forest. About 20% (426,479) of the total households in the city are in subnormal census tracts^b^.

To identify the cases of CD and SD in the city of Rio de Janeiro in 2011 and 2012, we used the complete database of the Notification Increase Information System (SINAN) of the Rio de Janeiro State Health Department.

During the years surveyed, 101,699 dengue cases were reported throughout the city of Rio de Janeiro, and 31,874 of those were confirmed by a laboratory. The database was cleaned, excluding duplicities with the help of the SPSS program’s merge function. A total of 30,751 cases were selected for this study.

The fields street and number were used for georeferencing. Data about the neighborhood available in the SINAN database refers to the neighborhood declared by the patient when he or she was filling the individual notification form (FIN) and, due to neighboring streets, it often does not represent the correct neighborhood of residence and is not a valid variable for georeferencing.

From the cases reported in SINAN, the georeferencing was performed using the automatic process of the ArcGis10 program’s Geocoding tool. This process consists in providing the latitude and longitude coordinates of each record by comparing it to a cartographic base. The cartographic base used was provided by the Núcleo de Geoprocessamento – ICICT/FIOCRUZ from the year 2000 on the scale 1:2,000. For the non-localized cases, we performed a manual georeferencing, which consisted of searching each address in Google Maps or the post office website. After the application of this technique, 17,466 records were georeferenced, i.e., about 57% of the total number of laboratory confirmed cases in 2011 and 2012, with 59% CD and 83% SD.

To verify if the loss occurred in CD cases influenced the spatial distribution obtained in relation to the SD cases, a loss simulation was performed in SD cases similar to the loss that occurred in CD cases. This simulation was carried out excluding from the database the difference between the percentage of severe cases in each neighborhood in relation to the classic georeferenced cases.

From the shapefile with CD and SD cases, spatial statistical analyses were performed, which allowed us to estimate their densities within the study area, using the Spatial Analysis extension of the ArcGis10 software for this calculation.

One of the analysis carried out was the kernel density estimator, and this study used the Gaussian function with a neighborhood radius of 900 m, defined automatically by the software. The intensity kernel allows us to estimate the number of events per unit area in each cell of a regular grid that covers the studied region^9^ ^,^ ^13^ ^,^ ^14^.

This non-parametric technique, in addition to estimating the intensity of occurrence of cases across the analyzed surface, allows us to filter the variability of a data set while retaining its main local characteristics^24^.

$$\hat{\lambda }\left(S\right)=\sum_{i=1}^{n}\frac{1}{\tau^{2}}\kappa \left(\frac{\left(s-\mathrm{si}\right)}{\tau }\right)$$

Explanation: λ (s) – intensity estimator; k ( ) – kernel weighting function; t – bandwidth; s – center of the area to be estimated; si – point location; n - total number of points (events).

In situations where the population is distributed heterogeneously in the space, the kernel density estimator map may not reflect the spatial distribution of the risk, so the kernel ratio was calculated using the logarithm (log) of the population living in the city of Rio de Janeiro. The transformation of the Napierian logarithm type into the population variable was made because some census tracts have zero population due to the lack of data availability from IBGE^16^, thus adjusting its distribution.

## RESULTS

**Figure 1:**
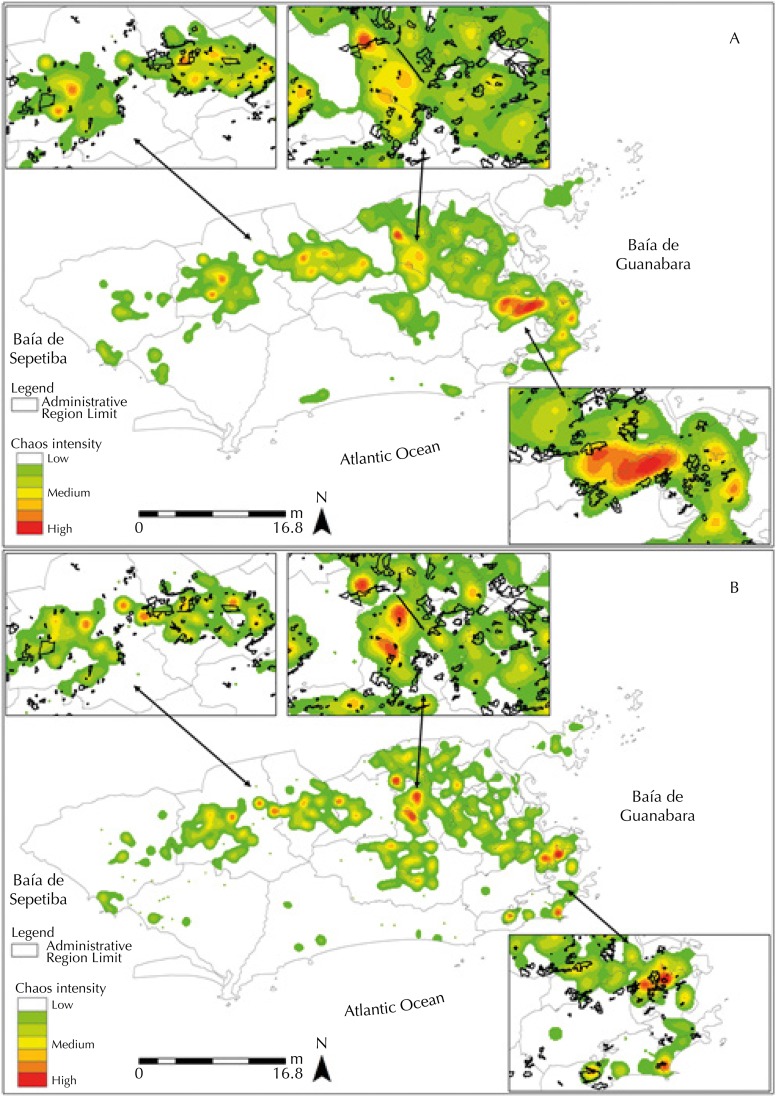
Kernel density of laboratory confirmed CD (A) and SD (B) cases in the city of Rio de Janeiro in the years 2011–2012.

Note: The rectangles highlighted in the figures are intended to expand areas that have high Kernel density. Localization information of the subnormal clusters was included in the highlighted rectangles, identified by black traces.

CD: classical dengue; SD: severe dengue

Although it is possible to observe a spatial distribution of cases throughout the territorial extension of the city, Figure 1 (A, B) shows that the Bangu, Vila Isabel, Tijuca, Anchieta, Madureira, and Campo Grande RA have CD hotspots (high-density areas). On the other hand, the Realengo, Jacarepaguá, Rio Comprido, Copacabana, Santa Teresa, Rocinha Anchieta, Madureira, and Campo Grande RAs have SD hotspots.

It should be noted that the Anchieta, Madureira, and Campo Grande RA have CD and SD hotspots and have a Social Development Index (SDI) lower than the one registered by the city of Rio de Janeiro.

**Figure 2:**
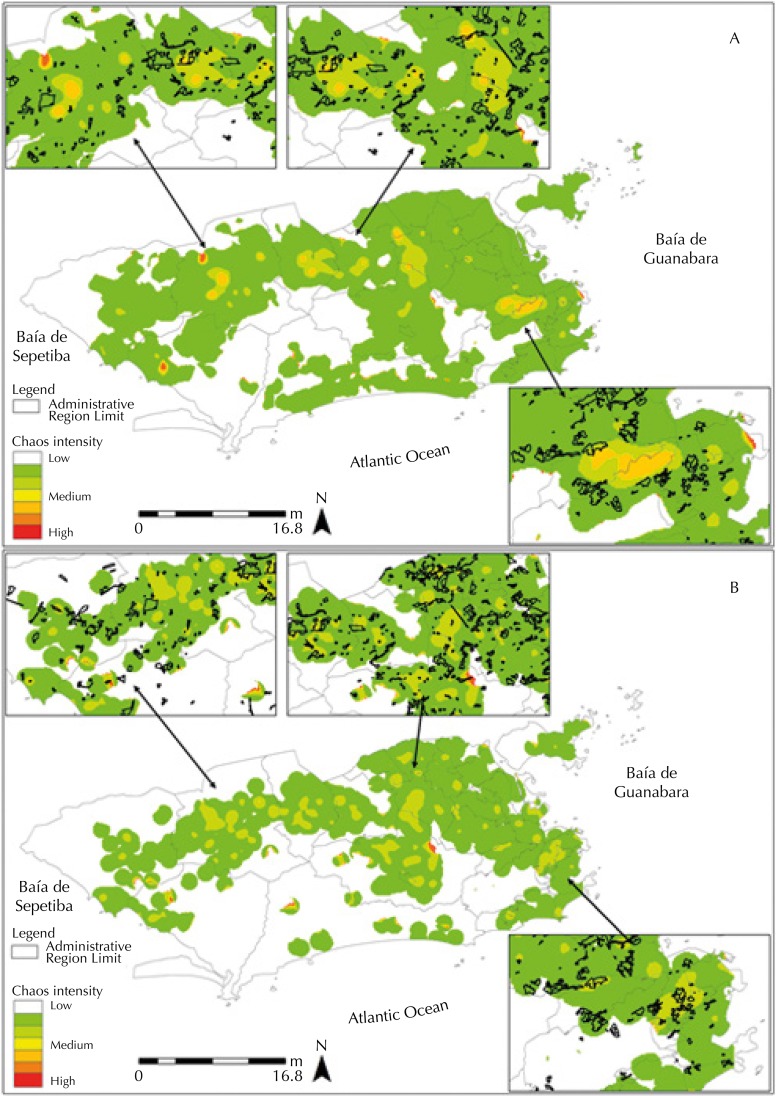
Kernel ratio of laboratory confirmed CD (A) and SD (B) cases in the city of Rio de Janeiro in the years 2011–2012.

Note: The rectangles highlighted in the figures are intended to expand areas that have high Kernel density. Localization information of the subnormal clusters was included in the highlighted rectangles, identified by black traces.

CD: classical dengue; SD: severe dengue

Figure 2 (A, B) shows CD hotspots in the Barra da Tijuca, Campo Grande, Centro, Guaratiba, Ilha do Governador, Jacarepaguá, Realengo, Santa Cruz, Santa Teresa, and Tijuca RA. When observing SD cases, the same RA have hotspots, except for the Tijuca neighborhood.

The highlighted rectangles in the figures show that the hotspots are inside or close to subnormal clusters (favelas).

Of the 33 RAs in the city of Rio de Janeiro, seven presented kernel densities between medium and high for CD cases and nine presented the same for SD cases. This shows that dengue is distributed geographically throughout the city, with a predominance of hotspots in severe cases.

The kernel ratio was calculated for CD and SD cases starting from the estimation of the density of cases and population, with an approximation of risk areas, since their values are weighted by the case-population relationship. The kernel ratio calculation was performed separately and showed a change in the pattern of spatial distribution found in kernel density, since the Anchieta, Bangu, Vila Isabel, Irajá, Madureira, Rio Comprido, Copacabana, and Rocinha RA no longer presented CD or SD hotspots.

The hotspots for classical dengue were observed in the administrative regions of Barra da Tijuca, Campo Grande, Centro, Guaratiba, Ilha do Governador, Jacarepaguá, Realengo, Santa Cruz, Santa Teresa, and Tijuca. The hotspots for severe dengue were found in Barra da Tijuca, Campo Grande, Centro, Guaratiba, Ilha do Governador, Jacarepaguá, Realengo, Santa Cruz, and Santa Teresa.

**Figure 3:**
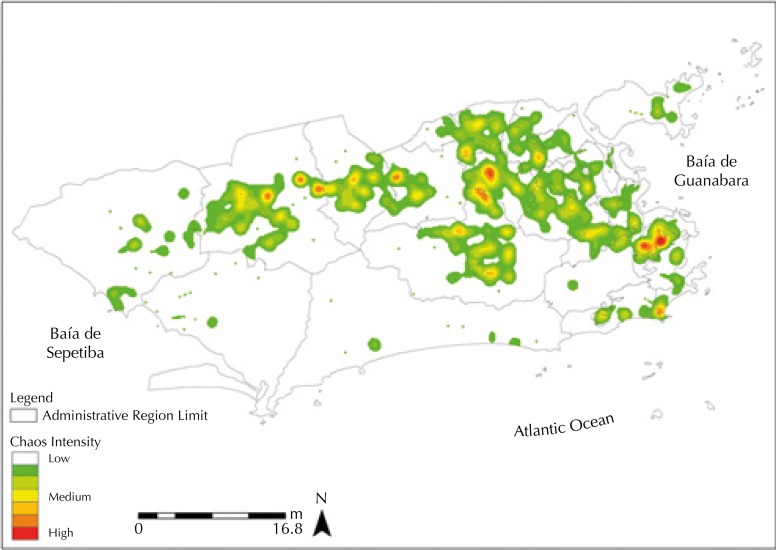
Kernel density of severe dengue cases in the city of Rio de Janeiro between 2011 and 2012, with a simulation of losses.

In the evaluation of the losses generated by the georeferencing process, there was a greater loss in CD records (41%) than in SD cases (17%), as observed in Figure 3. However, when we applied the simulation of losses in cases of severe dengue, the spatial distribution remained unchanged, not affecting the results presented previously.

## DISCUSSION

The city of Rio de Janeiro has different social and demographic characteristics, with different SDI for each of its administrative regions, the following of which have the lowest five indexes: Guaratiba (0.51); Complexo do Alemão, Jacarezinho, Rocinha, and Santa Cruz (0.54); Maré (0.55); Cidade de Deus and Pavuna (0.56); Bangu, Campo Grande, Portuária, and Vigário Geral (0.57)^b^.

It should be noted that the Anchieta, Bangu, Campo Grande, Jacarepaguá, Madureira, Rio Comprido, and Rocinha RA present hotspots in the kernel density and have an SDI below the SDI for Rio de Janeiro city (0.61).

The precarious sociodemographic condition, evidenced by the low SDI, leads the region to a process of structural violence, culminating in the oppression of groups, who are denied societal achievements, making them more vulnerable to suffering and death than others^19^. Violence threatens life, alters health, produces disease and make death a reality or a near possibility^1^.

As observed in Figures 1 and 2, the distribution of SD and CD cases does not coincide geographically throughout the territory, indicating that other factors, besides prior exposure to another serotype, may be associated with the aggravation of the case, such as social vulnerability^9^.

According to Flauzino et al.^12^, in a study carried out in the city of Niterói, RJ, hotspots, both in density and kernel ratio, in or near the favelas of Rio de Janeiro, suggest that the occurrence of dengue may be related to the social vulnerability of those populations. It is noteworthy that up to 2010, about 1,391,906 people lived in subnormal clusters (favelas) in the city of Rio de Janeiro^b^. 

Poor access to goods and services, such as education, leisure, work, culture, social health services, housing, water and sewage, as well as unemployment, inadequate work environment, low agricultural and food production^7^ contributes to the growth of this social vulnerability, reducing the individuals’ ability to respond to risk situations^15^ ^,^ ^20^. The violence often existing in these localities limits the actions of the government, contributing to this social vulnerability.

Figures 1 and 2 show that areas with higher density were in or near subnormal clusters, reinforcing the theory that socioeconomic aspects are also important to understand the spatial distribution of dengue^2^. Corroborating the findings, Chiaravalloti Neto et al.^9^ describe in their study the existence of a higher dengue risk in areas with higher demographic density and lower income.

The social organization in places of greater vulnerability directly implies health promotion actions. Among the objectives of these actions are improving urban infrastructures, such as basic sanitation, garbage collection, education, and health services, and to serve as a basis for the development of public policies that seek citizenship and quality of life^20^. This vulnerability can be minimized through interventions related to macroeconomic and job market policies, environmental protection, and the promotion of a culture of peace and solidarity^7^.

The lack of sanitary conditions favors the emergence of potential breeding places for *Aedes Aegypti*. The need to use water for body hygiene, the domicile, and even for food manipulation leads the population to store water in unfavorable or inappropriate containers, such as barrels, which cannot be fully sealed and facilitates the reproduction of the vector^5^ ^,^ ^8^ ^,^ ^25^.

The social organization will be related to the degree of empowerment of the population, since empowered individuals will have greater mobilization capacity with consequent optimization of existing resources to improve coping in risk situations^20^.

The inclusion of social inequalities as risk markers for dengue indicates the transformation of space and social dynamics as fundamental factors in the development of spaces conducive to the maintenance of dengue, and should, therefore, be prioritized in the public policy agenda^12^ ^,^ ^18^.

The application of the georeferencing technique and statistical analysis is important to evaluate the spatial distribution of dengue and to understand the factors that may be related to the occurrence of the cases. This technique allows the mapping of the disease and contributes to the structuring and analysis of social and environmental risks^23^, but the data stored in the information systems must be complete.

It is possible to highlight as a limitation of this study the losses registered in the georeferencing process, evidencing fragility in epidemiological surveillance actions, since it compromises the reading of the local reality and makes the decision-making process more difficult.

The results of this study suggest that barriers to access health services interfere with the early diagnosis of dengue, leading to a greater possibility of SD cases, since the late diagnosis of the disease may imply in failure to identify the warning signs of the disease. It should be noted that an improvement in access to health services is related to the principle of universality provided for in the Brazilian Unified Health System (SUS).

In this scenario, we can highlight that the Family Health Strategy (ESF) becomes a means to reduce the vulnerabilities in these areas. The ESF works with the identification of social determinants of health and the empowerment of communities to solve local problems, and contributes to correct the addresses provided during the first treatment, minimizing losses during the process of disease georeferencing.

The difference in loss of records between cases of CD and SD (Figure 3) can be attributed to the better completion of the individual investigation form (FII) in cases of SD. The severity of SD cases and the need to close them justifies this better filling. This fact indicates the need for permanent education to clarify the importance of completing the form also be mentioned in the cases of CD, in order to develop actions to monitor and control the aggravation.

It is noteworthy that, as losses occurred homogeneously in the analyzed territory, it did not include bias in the study.

The association between local inequalities and dengue incidence, using macroregions, such as the RA used in this study, does not allow for a detailed analysis of the internal heterogeneities existing in these spaces. This situation could be different with the use of census tracts as units of analysis. However, there are limitations to spatially locate population disease data obtained through the SINAN^4^ ^,^ ^5^ database, especially involving form filling faults^23^.

Another limitation of the study that must be highlighted is that, in epidemic periods, such as in the period under study, underreporting of CD may occur more frequently. In addition, the data filled in the Individual Notification Form (FIN) and Individual Investigation Form (FII) of the SINAN can poorer regarding the “street” field, causing the loss of records^24^.

The fight against dengue is not a simple task, since its occurrence, whether in the classical form or in the severe form, involves social factors and the provision of health services.

The spatial distribution analysis, using the Kernel estimator, is a valuable tool for planning actions, since it can and should be used to better understand the factors that contribute to the occurrence of dengue^3^.

The observation of social inequalities to understand the occurrence of CD and SD exposes the importance of geographic space and social dynamics in the definition of places with dengue maintenance capacity. This observation must be considered in the organization of strategic actions to combat the vector and in the planning of care provisions in areas of greater vulnerability, generating a positive impact in the control of the disease and reducing the severity of the disease.

## Footnotes

a Secretaria de Estado de Saúde do Rio de Janeiro, Subsecretaria de Vigilância em Saúde, Superintendência de Vigilância Epidemiológica e Ambiental.. Dengue: Estado do Rio de Janeiro. Rio de Janeiro; s.d. [cited 2017 Jun 20]. Available from: http://www.riocomsaude.rj.gov.br/Publico/MostrarArquivo.aspx?C=g3T65SJeoPk%3D

b Prefeitura Municipal do Rio de Janeiro, Instituto Pereira Passos. Armazém de dados. Rio de Janeiro; c2001 [cited 2017 Apr 6]. Available from: htpp://www.armazemdedados.rio.rj.gov.br/
